# Prevalence of Thyroid Disorder in a Primary Care District Hospital of Nepal

**DOI:** 10.31729/jnma.4240

**Published:** 2019-04-30

**Authors:** Priyanka Gupta, Pawan Kumar Bajaj Agrawal, Bikash Gauchan

**Affiliations:** 1 Department of Medical Services, Bayalpata Hospital, Achham, Nepal

**Keywords:** *hyperthyroidism*, *hypothyroidism*, *thyroid disorder*

## Abstract

**Introduction:**

Thyroid disorders are among the common endocrine disorders and may approximate diabetes in prevalence. District hospitals are in frontline to manage chronic disorders including thyroid. Primary care workforce of physicians and mid-level providers together deliver care in these hospitals. Few hospitals are equipped with tests to diagnose thyroid disorders. The objective of the study is to find the burden of thyroid disorder in a district hospital of Nepal.

**Methods:**

This was a descriptive cross sectional study conducted in Bayalpata Hospital. One year data from July 2017 to June 2018 was collected from the electronic health record system. Data was collected from 999 patients through convenient sampling where thyroid function test was done. Subgroup analysis was done on basis of gender, symptoms at presentation and comorbidities.

**Results:**

Prevalence of thyroid disorder in a district hospital of Nepal was 171 (17.11%) at 95% confidence interval, range occurring from 14% to 20%. Among them, 130 (76%) had hypothyroidism and 41 (24%) had hyperthyroidism. Prevalence of thyroid disorder among female was 147 (14.7%) and among male was 24 (2.4%). The most common symptom was depressed mood followed by nonspecific pain disorder, thyroid swelling, paresthesia and menstrual disturbances and common comorbidities reported were depression, diabetes, hypertension, anxiety disorder and chronic gastritis.

**Conclusions:**

Our study showed the burden of thyroid disorders in a primary care district hospital with **hypothyroidism being more common than hyperthyroidism.** Thyroid disorder must be addressed on time to lower the burden. However, most of the rural population of Nepal lack in matters of lack of resources. So, it is suggested for the need to equip the health centers with thyroid tests and integrated workforce of physicians and mid-level providers in care delivery of thyroid disorders.

## INTRODUCTION

Thyroid disorder are among the common endocrine disorders and may approximate diabetes in prevalence.^1,2^ Thyroid disorders are emerging as an evolving problem. Thyroid disorders can be classified based on the level of thyroid hormones in blood. Females are more commonly affected than males usually. Dietary iodine deficiency is an important cause for thyroid disorders.

Thyroid disorders may present with a wide range of clinical manifestations in the body. Sometimes no symptoms may be there as well in thyroid disorders. Hence it is necessary to train and equip integrated cohort of primary care physicians and mid-level health care providers to recognize and treat thyroid disorders and make appropriate referrals. District hospitals have been managing chronic diseases including endocrine disorders for a long time.^3,4^ Nevertheless we have limited data to reveal burden of thyroid disorder in primary careand to suggest quality of care.^5–7^

The aim of study is to find the burden of thyroid disorder in a rural district hospital of Nepal.

## METHODS

This was a descriptive cross-sectional study conducted in Bayalpata Hospital, a primary care district hospital in far west Nepal on patients attending outpatient department (OPD) over one year duration from July 2017 to June 2018. Due approval was sought from the hospital administration for access to the relevant data.

The sample size was calculated using formula


$$\text{n}={\text{(Z}}^{2}\times \text{p}\times \text{q)/}{\text{d}}^{2}=\text{​}{\left(1.96\right)}^{2}\times 0.5\times 0.5/{\left(0.05\right)}^{2}=\text{​}385$$

where,

n= sample size
Z= 1.96 with 95% confidence interval
p= 50%
d= 5%

The sample size calculated was 385 which were doubled for non-random sampling and final sample size was 770. The study used convenient sampling and included all patients in outpatient department in whom thyroid function test was done over one year duration with total of 999 patients. The data was extracted from electronic health record system of the hospital.^8^ As per the institutional protocol, TSH was measured in patients with symptoms of thyroid disorder and if TSH level was found beyond the normal range, free T3 and free T4 levels were measured in the same serum specimen. The normal ranges of TSH, free T3 & free T4 in the hospital laboratory were 0.39 to 6.16 /vIU/ml, 1.4 to 4.2 pg/ml and 0.8 to 2.2 ng/dl respectively.

Subgroup analysis was done on the basis of gender, symptoms at presentation and comorbities. The statistical analysis was performed using Statistical Package for the Social Sciences version 21.0 (IBM Inc., Armonk, New York, USA) and Microsoft Excel 2013 (Microsoft Corporation, Redmond, Washington, USA). Frequency and percentage were calculated for binary data.

## RESULTS

**Figure 1:**
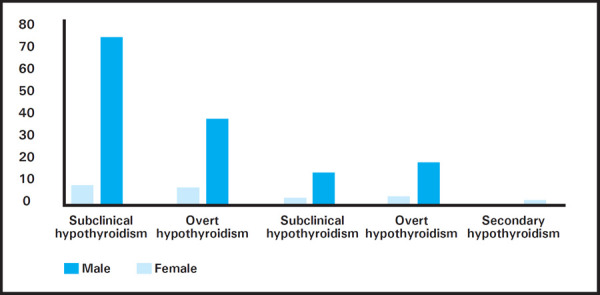
Gender distribution in thyroid disorders.

Thyroid function test was done in 999 patients with symptoms associated with thyroid disorder. Prevalence of thyroid disorder was 171 (17.11%) at 95% confidence interval, range occurring between 14% to 20%. Hypothyroidism was seen in 130 (76%) and hyperthyroidism was seen in 41 (24%) patients (Figure 1).

Among 999 patients, 799 were female and 200 were male. Prevalence of thyroid disorder among female and male of total sample population was 147 (14.7%) and 24 (2.4%) respectively. Similarly, 147 of 799 (18%) of female patients had thyroid disorder and 24 of 200 (20%) of male patients had thyroid disorder.

**Figure 2:**
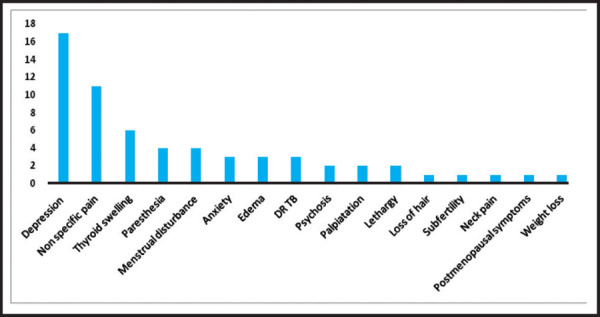
Presenting symptoms among new cases of thyroid dysfunction.

Depression was the most common presenting symptom followed by non-specific pain disorder, thyroid swelling, paresthesia and menstrual disturbances (Figure 2).

**Figure 3.**
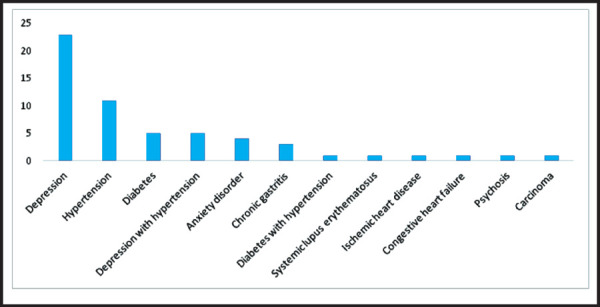
Comorbidities reported with thyroid dysfunction.

The comorbidities were reported in 57 (5.7%) cases. The most common comorbidity documented was depression, followed in order by hypertension, diabetes, anxiety disorder and chronic gastritis (Figure 3).

## DISCUSSION

Our study showed prevalence of thyroid disorder to be 171 (17.11%). Hypothyroidism was seen in 130 (76%) and hyperthyroidism was seen in 41 (24%) patients. Few district hospitals in suburban and rural Nepal have assimilated and analyzed their data on thyroid patients.^5–7^ and most of them fail to reflect upon their outcome for quality of care. We made an effort to describe thyroid disorder situation in rural district hospital.

With advances in laboratory investigations, there have been increasing dependence of healthcare personnel to rely on investigations because of limited interaction time between healthcare personnel and patients, fear of litigation and intention to improve diagnostic accuracy. A study in general practice in United Kingdom suggested that the thyroid function tests (TFTs) were prescribed in 12% patients and that the diagnostic yield of thyroid function test was 2.1%.^9^ This implicated the high use of TFTs in primary care patients which might be due to high number of non-specific symptoms at presentations. Our study showed that the thyroid disorders were more often suspected and diagnosed in females. It also showed that hypothyroidism was more common than hyperthyroidism. Our findings also revealed that in the spectrum of thyroid disorders, subclinical hypothyroidism was reported in highest number of patient.^2,5,6,9,10^

Depression, non-specific pain disorder, thyroid swelling, paresthesia and menstrual disturbances together contributed the majority of comorbidities associated with thyroid disorders. This was an important finding to guide the rational prescription of thyroid tests which would help to raise their diagnostic accuracy. This might be because of labor migrants who got diagnosed and later emigrated for employment opportunities in neighboring country India.^11^ The another possibility could be some of these patients were referred. Referral was not documented in electronic record and therefore was not reflected in this study. This might implicate both the low literacy rate in practice population of rural Nepal and thereby less understanding of the adherence to treatment and also the need to strengthen the knowledge of management of thyroid disorders in healthcare providers of this integrated cohort especially the mid-level providers. ^3,4^

Our study was based on a single hospital in rural Nepal and hence might not reflect the actual burden in rural community. The study relied on stored clinical data in the form of electronic record. Electronic record was considered better than papers in terms of legibility and durability. However, it might also have suffered the inadequate documentation of presenting symptoms, comorbidities and referrals as described previously.

The limitations of our study are that it cannot be generalized as non-probability sampling is done and the study has been done in small settings so the recommendations would be to study in large population settings with probability sampling so that it could be generalized.

## CONCLUSIONS

Our study showed the burden of thyroid disorders in district hospitals with hypothyroidism being more common than hyperthyroidism. Similarly, thyroid disorder was found to be more prevalent among females in comparison to males. Thyroid disorder must be addressed on time to lower the burden, however, most of the rural population of Nepal lack in matters of lack of resources so it is suggested for the need to equip the health centers with thyroid tests and integrated workforce of physicians and mid-level providers in care delivery of thyroid disorders to strengthen the quality, affordability and accessibility in primary care.
